# Treatment outcomes of stereotactic body radiation therapy for pulmonary metastasis from sarcoma: a multicenter, retrospective study

**DOI:** 10.1186/s13014-023-02255-y

**Published:** 2023-04-15

**Authors:** Tae Hoon Lee, Hak Jae Kim, Jin Ho Kim, Mi-Sook Kim, Won Il Jang, Eunji Kim, Kyung Su Kim

**Affiliations:** 1 Department of Radiation Oncology, Seoul National University Hospital, Seoul National University College of Medicine, 101, Daehak-ro, Jongno-gu, Seoul, 03080 Republic of Korea; 2 Cancer Research Institute, Seoul National University College of Medicine, Seoul, Republic of Korea; 3 Department of Radiation Oncology, Korea Institute of Radiological and Medical Sciences, 75, Nowon-ro, Nowon-gu, Seoul, 01812 Republic of Korea; 4 Department of Radiation Oncology, Seoul Metropolitan Government-Seoul National University Boramae Medical Center, 20, Boramae-ro 5 Gil, Dongjak-gu Seoul, 07061 Republic of Korea

**Keywords:** Sarcoma, Stereotactic body radiotherapy, Oligometastasis, Oligoprogression, Radiation dose fractionation

## Abstract

**Purpose:**

The aim of this study was to evaluate the treatment outcomes and potential dose-response relationship of stereotactic body radiation therapy (SBRT) for pulmonary metastasis of sarcoma.

**Materials and methods:**

A retrospective review of 39 patients and 71 lesions treated with SBRT from two institutions was performed. The patients had oligometastatic or oligoprogressive disease, or were receiving palliation. Doses of 20–60 Gy were delivered in 1–5 fractions. The local control per tumor (LCpT) was evaluated according to the biologically effective dose with an α/β ratio of 10 (BED_10_) of the prescribed dose (BED_10_ ≥ 100 Gy vs. BED_10_ < 100 Gy). Clinical outcomes per patient, including local control per patient (LCpP), pulmonary progression-free rate (PPFR), any progression-free rate (APFR), and overall survival (OS) were investigated.

**Results:**

The median follow-up period was 27.2 months. The 1-, 2-, and 3-year LCpT rates for the entire cohort were 100.0%, 88.3%, and 73.6%, respectively. There was no observed difference in LCpT between the two BED_10_ groups (*p* = 0.180). The 3-year LCpP, PPFR, APFR, and OS rates were 78.1%, 22.7%, 12.9%, and 83.7%, respectively. Five (12.8%) patients with oligometastasis had long-term disease-free intervals, with a median survival period of 40.7 months. Factors that were associated with a worse prognosis were oligoprogression (vs. oligometastasis), multiple pulmonary metastases, and simultaneous extrathoracic metastasis.

**Conclusion:**

SBRT for pulmonary metastasis of sarcoma is effective. Some selected patients may achieve durable response. Considerations of SBRT indication and disease extent may be needed as they may influence the prognosis.

**Supplementary Information:**

The online version contains supplementary material available at 10.1186/s13014-023-02255-y.

## Introduction

Sarcoma is a malignancy that originates from mesenchymal cells and accounts for 1% of adult malignancies [1]. There are numerous subtypes of sarcoma with different clinical behaviors [2], and the rarity and heterogeneity of the disease can make it difficult to access appropriate treatment approaches. Many patients with sarcoma eventually experience hematogenous spread, which primarily involves the lungs [3]. Local therapies for metastatic lesions can be a feasible treatment option when the number of metastatic lesions is limited. Stereotactic body radiation therapy (SBRT) is an effective treatment modality for this approach. Long-term results from the SABR-COMET trial have reported a survival benefit of SBRT for metastatic tumors with a limited number [4]. The lungs are one of the most feasible sites for SBRT, and lung tumors, including primary lung tumors and metastatic tumors from various primary sites, are being treated with SBRT.

Local ablative strategies for pulmonary metastasis of sarcoma are feasible. Surgical resection of metastatic lesions of sarcoma may provide long-term survival for selected patients [5, 6]. Historically, sarcoma was considered a relatively radioresistant tumor [7], and radiation therapy was used in limited purpose. However, with recent technical advancements, SBRT is now used for local ablative treatment of metastatic lesions of sarcoma [8]. Several previous studies have reported high local control rates and tolerable toxicities when treating pulmonary metastatic tumors of sarcoma using SBRT [9, 10]. However, optimal dose-fractionation schemes for SBRT for pulmonary metastatic lesions of sarcoma have not yet been established. Studies analyzing SBRT for primary lung tumors and pulmonary metastasis of other histologies have concluded that dose escalation may increase local control rates, but the potential risk of increased toxicities is a concern [11, 12]. The purpose of this study was to report treatment outcomes of SBRT with various dose-fractionation schemes for pulmonary metastasis of sarcoma and to provide insights for selecting appropriate dose-fractionation schemes for different clinical situations.

## Materials and methods

### Patient population

The medical records of patients with pulmonary metastatic lesions of sarcoma treated with SBRT at two institutions (Seoul National University Hospital and Korea Cancer Center Hospital) between January 2011 and December 2021 were retrospectively reviewed. Cases of pulmonary metastasis of carcinoma with sarcomatoid change or sarcomatoid features, metastasis from a primary site of soft tissue that was not of mesenchymal cell origin, and tumors without follow-up computed tomography (CT) scans were excluded from the study. A total of 71 pulmonary metastatic lesions from 39 patients treated with SBRT were eligible.

### Treatment

Patients with pulmonary metastasis of sarcoma were referred by treating surgeons or medical oncologists to the radiation oncology department for local ablation of metastatic tumors. There were three categories of indications for SBRT: (1) Oligometastasis, defined as a limited number (≤ 5) of metastatic lesions at diagnosis or recurrence; (2) Oligoprogression, defined as a limited number (≤ 5) of metastatic lesions that progressed or did not respond during systemic therapy or within 6 months after the completion of systemic therapy; (3) Palliation, defined as metastatic disease that is not under systemic therapy, with numerous active metastatic lesions, and some rapidly progressive tumors that require local treatment. The treating radiation oncologist re-evaluated the feasibility of SBRT for pulmonary lesions and determined the necessity of treating such lung nodules.

For radiation therapy planning, CT-based simulation was performed. The patient was placed in a supine position with both arms abducted, using a wing board and vacuum cushions for immobilization. An abdominal compression plate was used to limit the movement of the diaphragm during breathing. For metastatic nodules located in the lung apex, the patient was placed in a supine position with both arms adducted and immobilized using thermoplastic aquaplast. Four-dimensional CT was performed to generate CT images for each respiratory phase. The internal target volume (ITV) was delineated by the radiation oncologist based on these CT images, taken at different respiratory phases. The planning target volume (PTV) was generated by expanding the ITV by 3–7 mm. The prescribed dose and fractionation were determined by the radiation oncologist, taking into account the indication for SBRT, proximity of the lung nodules to critical mediastinal structures, and the patient’s baseline lung function.

The radiation dose was delivered to patients using various devices. Radiation therapy was delivered mainly using TrueBeam and Clinac iX (Varian Medical Systems Inc., Palo Alto, CA, USA). Volumetric modulated arch therapy was applied for radiation therapy planning and beam delivery using these devices. For patients with large respiratory motion, treatment was delivered using MRIdian (ViewRay Inc., Oakwood Village, OH, USA), a device that utilizes magnetic resonance imaging-based gating. For patients with limited lung function and appropriate for tracking gold fiducial markers, treatment was delivered using CyberKnife (Accuray Inc., Sunnyvale, CA, USA). Volume-based prescriptions were used for radiation dose prescriptions in all four devices. For CyberKnife, the dose was prescribed to the 75% isodose line, and the maximum dose reached around 130% of the prescribed dose. For the other devices, the plan was optimized to cover 95% of the PTV by 100% of prescribed dose, and the maximum dose was limited to around 110% of the prescribed dose. Each fraction was delivered two or three times per week without consecutive daily treatment. Treatment of multiple pulmonary lesions varied based on their proximity to one another. When lung nodules were far apart, each one was treated with an individual plan, which were delivered either on the same day, on alternate days, or consecutively. Lesions close to each other were treated with a single isocenter plan.

### Endpoints and statistics

The primary endpoint of this study was local control per tumor (LCpT). An event of LCpT was defined as regrowth of the treated tumor or the formation of new lesions inside the PTV. LCpT was measured from the start of SBRT to the lesion, and calculated using the Kaplan-Meier method for each pulmonary metastatic tumor. The biologically effective dose with an α/β ratio of 10 (BED_10_) was calculated for each lung tumor using the following equation:

$${\text{BED}}_{{10}} = nd\left( {1 + \frac{d}{{\upalpha /\upbeta }}} \right)$$

where *n* is the number of fractions, *d* is the dose per fraction in Gy, and α/β is the dose at which the linear and quadratic portions of cell killing are equal in the linear-quadratic model. Prescribed dose was used for the calculation of BED_10_. Treated lung tumors were categorized into two groups: a low BED_10_ group, defined by BED_10_ < 100 Gy, and a high BED_10_ group, defined as BED_10_ ≥ 100 Gy. Although there were reports with lower α/β ratios for sarcoma [13–15], an α/β ratio of 10 and a BED_10_ cutoff value of 100 Gy were chosen based on previous literature for SBRT for primary lung tumors and metastatic lung tumors [16, 17]. Comparison of LCpT between the two groups was performed using a log-rank test. The centrality of treated lung nodules was defined in two ways: using conventional Radiation Therapy Oncology Group criteria, which defined a central nodule as a location within 2 cm from the proximal bronchial tree[18], and by abutment to the mediastinal structures such as the heart, great vessels, and trachea. The response of the treated lesion at the last follow-up was evaluated per tumor based on the Response Evaluation Criteria in Solid Tumors.

The observed clinical outcomes per patient were local control per patient (LCpP), pulmonary progression-free rate (PPFR), any progression-free rate (APFR), and overall survival (OS). An event for LCpP was defined as any LCpT event for lesions treated with SBRT for the patient. An event for PPFR was defined as the progression of any metastatic lesions in the lung. An event for APFR was defined as the progression of any intra- and extra-thoracic metastatic lesions. An event for OS was defined as the death of the patient from any cause. These clinical outcomes per patient were measured from the start of the first SBRT session and calculated using the Kaplan-Meier method. Comparison of these clinical outcomes between the two groups was not possible as some patients with multiple pulmonary metastatic lesions underwent simultaneous SBRT sessions with different BED_10_.

Univariate analyses were performed on LCpT, LCpP, PPFR, APFR, and OS to search for variables associated with corresponding endpoints. The Cox proportional hazards model was used for the univariate analysis. When no event occurred with a specific variable, the hazard ratio could not be derived, and the p-value was calculated using the log-rank test instead. Multivariate analysis was not performed due to a low number of cases and events. SBRT-related adverse events were graded using the Common Terminology Criteria for Adverse Events version 5, and severe (grade ≥ 3) adverse events were reported. Student’s t-test was used for the comparison of continuous variables, and chi-square test with or without Yate’s continuity correction was used for the comparison of categorical variables. A p-value less than 0.05 was defined as statistically significant. All statistical analyses were performed using R 4.2.1 (The R Foundation for Statistical Computing, Vienna, Austria).

## Results

### Patient and tumor characteristics

The median follow-up period from the initiation of SBRT treatment was 27.2 months (range, 1.1–69.4 months) per patient and 19.2 months (range, 1.1–69.4 months) per tumor. The patient characteristics of 39 eligible patients are summarized in Table 1. More than half (61.5%) of the patients had a primary site of extremities. The three most frequent histologies were leiomyosarcoma (23.1%), osteosarcoma (17.9%), and undifferentiated pleomorphic sarcoma (12.8%). The other 46.2% consisted of 9 different histologies, indicating the heterogeneity of the cohort. All patients with reported histologic grade had grade 2–3. Histologic grade could not be retrieved for 7 (17.9%) patients, as they were referred in the middle of their disease course without a detailed pathologic report. Almost half (51.3%) of the patients had multiple pulmonary metastatic lesions at the time of disease recurrence. 48.7% of the patients underwent multiple SBRT sessions within the follow-up period. Five (12.8%) patients had no evidence of disease in the last follow-up visit. All patients with no evidence of disease in the last follow-up had SBRT indication of oligometastasis, and four of them presented with a single pulmonary metastatic lesion. The median survival period of these patients from the initiation of SBRT was 40.7 months (range, 19.0–55.9 months). Three patients who achieved no evidence of disease underwent a single course of SBRT, while the other two patients underwent 2 and 3 courses of SBRT each.

**Table 1:** Patient characteristics

Characteristics	Numbers (N = 39)
Age (median, years)	59 (range, 19–86)
*Sex*	
Male	21 (53.8%)
Female	18 (46.2%)
*ECOG Performance status at radiation oncology department presentation*	
0–1	18 (46.2%)
2	7 (17.9%)
Not reported	14 (35.9%)
*Primary site*	
Lower extremity	19 (48.7%)
Upper extremity	5 (12.8%)
Uterus	5 (12.8%)
Chest wall	2 (5.1%)
Lung	2 (5.1%)
Retroperitoneum	2 (5.1%)
Buttock	1 (2.6%)
Head and neck	1 (2.6%)
Pelvis	1 (2.6%)
Stomach	1 (2.6%)
*Histology*	
Leiomyosarcoma	9 (23.1%)
Osteosarcoma	7 (17.9%)
Undifferentiated pleomorphic sarcoma	5 (12.8%)
Liposarcoma	4 (10.3%)
Synovial sarcoma	4 (10.3%)
Chondrosarcoma	2 (5.1%)
Myxofibrosarcoma	2 (5.1%)
Spindle cell sarcoma	2 (5.1%)
Alveolar soft part sarcoma	1 (2.6%)
Ewing sarcoma	1 (2.6%)
Malignant glomus tumor	1 (2.6%)
Malignant peripheral nerve sheath tumor	1 (2.6%)
*Histological grade*	
Not reported	7 (17.9%)
2	13 (33.3%)
3	19 (48.7%)
History of surgery at primary site	38 (97.4%)
Distant metastasis at initial diagnosis	6 (15.4%)
*Number of lung lesions at recurrence*	
1	19 (48.7%)
2	8 (20.5%)
3	4 (10.3%)
Numerous	8 (20.5%)
Simultaneous extrathoracic metastasis	16 (41.0%)
*Indication of the first SBRT session*	
Oligometastasis	23 (59.0%)
Oligoprogression	10 (25.6%)
Palliation	6 (15.4%)
*Progression-free interval*	
≤ 1 year	29 (74.4%)
> 1 year	10 (25.6%)
*Total number of treated lung lesions by SBRT*	
1	20 (51.3%)
2	8 (20.5%)
3	6 (15.4%)
4	4 (10.3%)
5	1 (2.6%)
*Patient status at last follow-up*	
Alive with disease	29 (74.4%)
No evidence of disease	5 (12.8%)
Died of disease	5 (12.8%)

*ECOG* Eastern Cooperative Oncology Group; *SBRT* Stereotactic body radiation therapy

Characteristics of the tumors and treatments according to the BED_10_ groups were summarized in Table 2. The median follow-up period per tumor was 19.3 months (range, 4.7–67.9 months) in the low BED_10_ group and 21.9 months (range, 1.1–69.4 months) in the high BED_10_ group, without a statistically significant difference (*p* = 0.418). Additional file 1: Table S1 summarized the dose-fractionation regimens used in SBRT courses and their BED_10_ and BED_4_. A wide range of prescribed BED_10_ was reported (40–180 Gy). Several differences were observed between the two groups. There were more patients who underwent palliative SBRT (48.3% vs. 4.8%) in the low BED_10_ group than in the high BED_10_ group, and more patients had oligometastatic disease in the high BED_10_ group than in the low BED_10_ group (66.7% vs. 27.6%). Simultaneous extrathoracic metastasis were more frequent in the low BED_10_ group than in the high BED_10_ group (69.0% vs. 31.0%). This difference was presumably due to the difference in SBRT indication between the two groups. There was more stable disease in response of treated lesion in the low BED_10_ group than in the high BED_10_ group (34.5% vs. 7.1%). No difference was observed in the diameter of metastatic lesions, the size of the ITV, the size of the PTV, or the histologic grade.

**Table 2:** Characteristics of tumors and treatments according to the biologically effective dose

Characteristics	Low BED group (BED_10_ < 100 Gy, N = 29)	High BED group (BED_10_ ≥ 100 Gy, N = 42)	*p* value
Diameter of metastatic lesion (median, cm)	1.2 (range, 0.3–3.6)	1.3 (range, 0.4–3.3)	0.686
*Location of metastatic lesion*			
Left upper lobe	6 (20.7%)	8 (19.0%)	0.228
Left lower lobe	7 (24.1%)	11 (26.2%)	
Right upper lobe	3 (10.3%)	10 (23.8%)	
Right middle lobe	6 (20.7%)	2 (4.8%)	
Right lower lobe	7 (24.1%)	11 (26.2%)	
*Centrality*			
RTOG criteria	2 (6.9%)	6 (14.3%)	0.031
Abutment to the mediastinal structure	6 (20.7%)	1 (2.4%)	
None	21 (72.4%)	35 (83.3%)	
*Histological grade*			
2	4 (13.8%)	14 (33.3%)	0.308*
3	16 (55.2%)	24 (57.1%)	
Unknown	9 (31.0%)	4 (9.5%)	
*Indication of SBRT*			
Oligometastasis	8 (27.6%)	28 (66.7%)	< 0.001
Oligoprogression	7 (24.1%)	12 (28.6%)	
Palliative	14 (48.3%)	2 (4.8%)	
Single pulmonary lesion at recurrence	6 (20.7%)	21 (50.0%)	0.024
Simultaneous extrathoracic metastasis	20 (69.0%)	13 (31.0%)	0.004
*Progression-free interval*			
≤ 1 year	26 (89.7%)	31 (73.8%)	0.178
> 1 year	3 (10.3%)	11 (26.2%)	
Total prescribed dose of SBRT (median, Gy)	30 (range, 20–44)	54 (range, 45–60)	< 0.001
*Number of fractions of SBRT*			
1	4 (13.8%)	0 (0.0%)	< 0.001
2	4 (13.8%)	1 (2.4%)	
3	10 (34.5%)	4 (9.5%)	
4	10 (34.5%)	34 (81.0%)	
5	1 (3.4%)	3 (7.1%)	
BED_10_ (median, Gy)	75.0 (range, 40.0–95.2)	126.9 (range, 100.0–180.0)	< 0.001
Size of internal target volume (median, cm^3^)	1.4 (range, 0.2–29.1)	2.4 (range, 0.4–29.3)	0.804
Size of planning target volume (median, cm^3^)	6.2 (range, 2.0–67.7)	10.9 (range, 1.4–52.9)	0.587
*Response of treated lesion at the last follow-up*			
Complete response	17 (58.6%)	25 (59.5%)	0.004
Partial response	2 (6.9%)	7 (16.7%)	
Stable disease	10 (34.5%)	3 (7.1%)	
Progressive disease	0 (0.0%)	7 (16.7%)	

* Calculated without cases with unknown value

BED_10_: Biologically effective dose with an α/β ratio of 10; RTOG: Radiation Therapy Oncology Group; SBRT: Stereotactic body radiation therapy

### Clinical outcomes

**Fig. 1:**
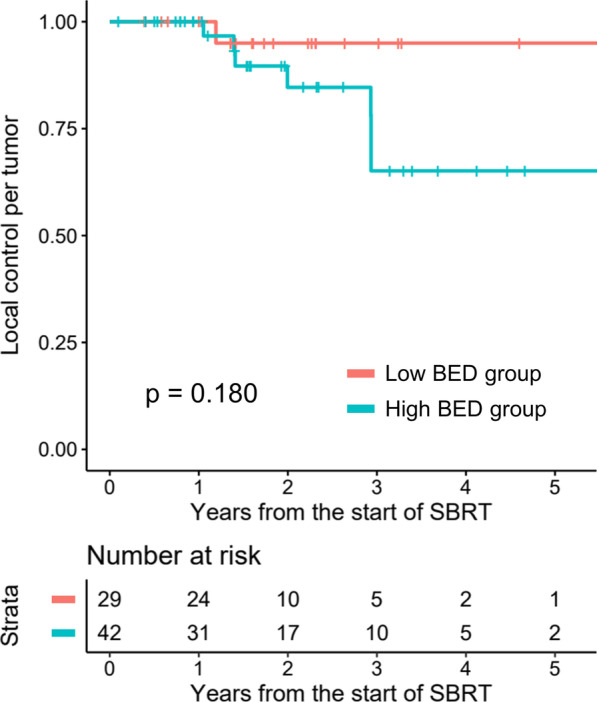
The actuarial rate of local control per tumor according to the biologically effective dose (BED) group

A total of 8 LCpT events were reported. Three LCpT events occurred in one patient with leiomyosarcoma, who experienced recurrences after systemic treatment at a similar time period. The 1-, 2-, and 3-year LCpT rates for all tumors were 100.0%, 88.3%, and 73.6%, respectively. The actuarial rate of LCpT according to the BED_10_ groups was illustrated in Fig. 1. The 1-, 2-, and 3-year LCpT rates were 100.0%, 95.0%, and 95.0% for the low BED_10_ group, respectively, and 100.0%, 84.6%, and 65.1% for the high BED_10_ group, respectively. No statistically significant difference was observed between the two groups (*p* = 0.180). Results of the univariate analysis for LCpT were summarized in Table 3. No variables were found to be associated with LCpT in the univariate analysis.

**Table 3:** Univariate analysis for local control per tumor

Characteristics (comparison vs. reference)	Hazard ratio	95% confidence interval	*p* value
BED category (BED_10_ ≥ 100 Gy vs. BED_10_ < 100 Gy)	3.812	0.467–31.13	0.212
Diameter of metastatic lesion (continuous, per cm)	1.383	0.557–3.436	0.484
Location of metastatic lesion (right vs. left)	5.869	0.721–47.75	0.098
Central nodule by RTOG criteria (yes vs. no)	1.610	0.197–13.18	0.657
Abutment to the mediastinal structure (yes vs. no)	No event for “yes”		0.321*
Extremity as primary site (yes vs. no)	0.276	0.055–1.377	0.117
Histological grade^†^ (grade 3 vs. grade 2)	2.853	0.528–15.41	0.223
Distant metastasis at initial diagnosis (yes vs. no)	No event for “yes”		0.639*
Indication of SBRT			
Oligoprogression vs. oligometastasis	2.493	0.551–11.27	0.235
Palliation vs. oligometastasis	0.773	0.084–7.115	0.820
Single pulmonary lesion at recurrence (yes vs. no)	0.711	0.170–2.983	0.641
Simultaneous extrathoracic metastasis (yes vs. no)	2.885	0.679–12.26	0.151
Progression-free interval (> 1 year vs. ≤ 1 year)	0.213	0.025–1.786	0.154

* This* p* value was calculated using log-rank test

^†^ Calculated without cases with unknown value

BED_10_: Biologically effective dose with an α/β ratio of 10; RTOG: Radiation Therapy Oncology Group; SBRT: Stereotactic body radiation therapy

**Fig. 2:**
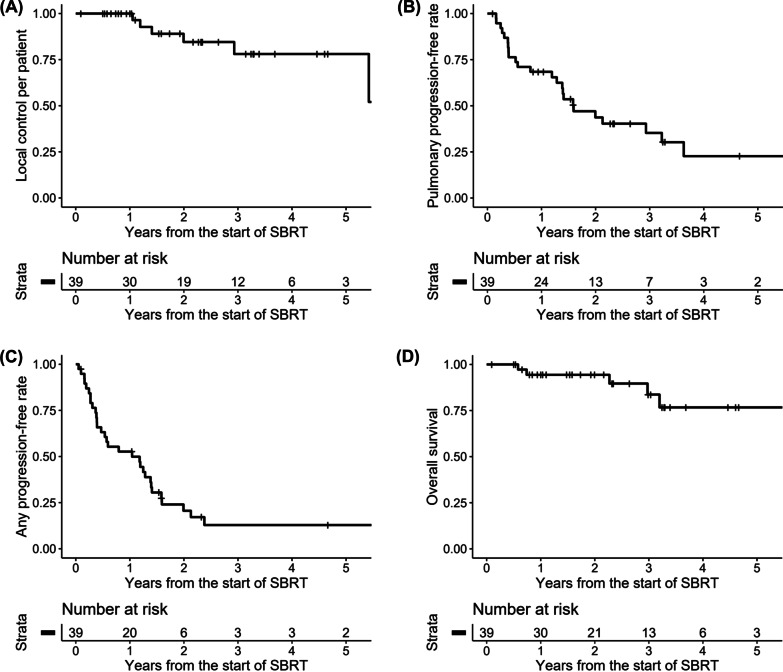
The actuarial rate of **A** local control per patient, **B** pulmonary progression-free rate, **C** any progression-free rate, and **D** overall survival

There were 6 patients with in-field local recurrence, 23 patients with out-field intrathoracic recurrence, and 29 patients with extrathoracic recurrence. A total of 6 LCpP events, 24 PPFR events, 31 APFR events, and 5 OS events were reported. The actuarial rates of clinical outcomes per patient were illustrated in Fig. 2. The 1-, 2-, and 3-year LCpP rates were 100.0%, 84.6%, and 78.1%, respectively, and the 1-, 2-, 3-year PPFR were 68.4%, 43.7%, and 22.7%, respectively. The 1-, 2-, and 3-year APFR were 52.7%, 20.6%, and 12.9%, respectively, and the 1-, 2-, 3-year OS rates were 94.4%, 94.4%, and 83.7%, respectively. Results of the univariate analysis for clinical outcomes per patient were summarized in Table 4. Simultaneous extrathoracic metastasis was significantly associated with worse APFR [hazard ratio (HR) 3.184, 95% confidence interval (CI) 1.474–6.878,* p* = 0.003] and OS (HR 9.953, 95% CI 1.072–92.39,* p* = 0.043). Oligoprogression (vs. oligometastasis) was significantly associated with worse OS (HR 14.48, 95% CI 1.444–145.3,* p* = 0.023). No OS events were reported for patients with a single pulmonary lesion at recurrence, and a significant association of this variable with better prognosis was found (log-rank* p* = 0.017).

**Table 4:** Univariate analysis for treatment outcomes per patient

Characteristics (comparison vs. reference)	Local control per patient			Pulmonary progression-free rate			Any progression-free rate			Overall survival		
	HR	95% CI	*p* value	HR	95% CI	*p* value	HR	95% CI	*p* value	HR	95% CI	*p* value
Age (continuous, per year)	0.980	0.935–1.026	0.387	0.995	0.974–1.017	0.671	0.996	0.978–1.015	0.689	0.981	0.932–1.033	0.470
ECOG Performance status* (2 vs. 0–1)	0.590	0.061–5.682	0.648	0.804	0.251–2.576	0.714	0.952	0.334–2.720	0.927	No event for “2”		0.508*
Extremity as primary site (yes vs. no)	0.366	0.066–2.014	0.248	1.208	0.526–2.775	0.656	1.022	0.495–2.111	0.953	0.502	0.083–3.025	0.452
Histological grade^†^ (grade 3 vs. grade 2)	3.546	0.360–34.88	0.278	1.860	0.726–4.766	0.196	1.403	0.630–3.124	0.407	1.773	0.284–11.09	0.540
Distant metastasis at initial diagnosis (yes vs. no)	No event for “yes”		0.646*	1.768	0.515–6.071	0.365	2.616	0.937–7.306	0.067	4.593	0.394–53.55	0.224
Indication of SBRT												
Oligoprogression vs. oligometastasis	1.398	0.145–13.48	0.772	2.291	0.946–5.550	0.066	2.221	0.983–5.017	0.055	14.48	1.444–145.3	**0.023**
Palliation vs. oligometastasis	1.282	0.129–12.70	0.832	0.506	0.114–2.246	0.370	1.897	0.685–5.255	0.218	4.956	0.301–81.72	0.263
Single pulmonary lesion at recurrence (yes vs. no)	0.544	0.237–1.250	0.152	0.544	0.237–1.250	0.152	0.552	0.269–1.130	0.104	No event for “yes”		0.017*
Simultaneous extrathoracic metastasis (yes vs. no)	1.668	0.730–3.811	0.225	1.668	0.730–3.811	0.225	3.184	1.474–6.878	**0.003**	9.953	1.072–92.39	**0.043**
Progression-free interval (> 1 year vs. ≤ 1 year)	0.489	0.178–1.338	0.163	0.489	0.178–1.338	0.163	0.465	0.199–1.089	0.078	0.368	0.040–3.372	0.376

*P*-values less than 0.05 were marked as bold

* This* p* value was calculated using log-rank test

^†^ Calculated without cases with unknown value

*CI* Confidence interval; *HR* Hazard ratio; *ECOG* Eastern Cooperative Oncology Group; *SBRT* Stereotactic body radiation therapy

One patient reported grade 3 radiation pneumonitis as an adverse event. This patient was diagnosed with spindle cell sarcoma of the lung and underwent multiple resections of pulmonary lesions. After these resections, the patient received a total of five courses of radiation therapy, and four of these treatment courses were SBRT. All SBRT plans had BED_10_ over 100 Gy. Five years after the initial SBRT course and 11 months after the last SBRT course, the patient required oxygen therapy due to radiation pneumonitis and fibrosis.

## Discussion

This study observed the effectiveness of SBRT for pulmonary metastases of sarcoma. The 3-year LCpT and LCpP rates for the whole cohort was 73.6% and 78.1%, respectively. Several previous studies, both prospective and retrospective, have reported treatment outcomes of SBRT for pulmonary metastases of sarcoma. Some selected studies are summarized in Table 5 [9, 19–25]. These previous studies have reported high LCpT rates ranging from 82 to 100%. Sarcoma was traditionally considered a relatively radioresistant tumor [7], but the reported LCpT rates of SBRT for pulmonary metastases of sarcoma in the literature were not particularly low compared to the LCpT rates of SBRT for primary lung tumors [26], which are around 90% [27]. This may be attributed to the large radiation dose per fraction of SBRT. Sarcoma has a relatively low α/β ratio, meaning that sarcoma is more sensitive to fraction size than other types of tumors [13, 28]. The current study showed a relatively low rate of LCpT. It should be noted that 3 of the 8 LCpT events in this study occurred in a single patient. The disease characteristics of this particular patient largely affected the outcome due to the small size of the patient cohort. In general, SBRT for pulmonary metastases of sarcoma is effective for local disease control. For patients with a limited number of metastatic lesions, controlling the metastatic lesions can provide prognostic benefit[4]. Although the patient cohort from the landmark study of SABR-COMET mostly consisted of other tumor histologies, it can be presumed that sarcoma patients with a limited number of metastatic lesions may also benefit from treating metastatic lesions with SBRT. In the current study, five (12.8%) patients with oligometastatic disease eventually achieved no evidence of disease with lung SBRT. These patients clearly benefited from the application of SBRT for the pulmonary metastatic lesions.

**Table 5:** Outcomes of stereotactic body radiotherapy for pulmonary metastasis of sarcoma from selected previous studies

Study	Design	Time period	Number of patients	Number of tumors	Dose	Number of fractions	Median follow-up	Local control per tumor	Overall survival	Severe toxicity
Dhakal et al. [19]	Retrospective (SBRT vs. surgery)	1990–2006	15 from total 52	74	30–55 Gy (50–55 Gy: 62%)	10	11 months	2-year 88%, 3-year 82%	Median 2.1 years	No
Mehta et al. [20]	Retrospective	2009–2011	16	25	36–54 Gy (50–54 Gy: 88%)	3–4	20 months	94% at 43 months	4-year 72%	No
Frakulli et al. [21]	Retrospective	2010–2014	24	68	30–60 Gy	3–8	17 months	1-year 88.2%, 2-year 85.9%	1-year 73.1%, 2-year 66.4%	No
Navarria et al. [22]	Prospective observational	2008–2014	28	51	30 Gy in 1 fx, 60 Gy in 3 fx, 48 Gy in 4 fx, 60 Gy in 8 fx		21 months	5-year 96%	2-year 55.7%, 5-year 43.3%	No
Soyfer et al. [23]	Retrospective	2009–2013	22	53	21 Gy in 3 fx, 40 Gy in 4 fx, 48 Gy in 4 fx, 60 Gy in 3 fx		95 months from diagnosis	100%	5-year 50%	One grade 3 pulmonary toxicity
Lindsay et al. [24]	Retrospective	2005–2014	44	117	30–55 Gy (50–55 Gy: 75%)	5–12 (10: 75%)	14 months	Crude rate 95%	2-year 82%, 5-year 50%	One esophageal stricture
Baumann et al. [9]	Retrospective (multi-institution)	2011–2016	44	56	24–50 Gy (50 Gy: 80%)	3–5 (4–5: 94%)	16 months	1-year 96%, 2-year 90%	1-year 74%, 2-year 46%	No
Navarria et al. [25]	Prospective phase II	2015–2020	44	71	30 Gy in 1 fx, 60 Gy in 3 fx, 48 Gy in 4 fx, 60 Gy in 8 fx		48 months	1-year 98.5%, 2-year 98.5%, 3-year 98.5%, 4-year 93.1%, 5-year 93.1%	1-year 88.6%, 2-year 66.7%, 3-year 56.8%, 4-year 53.0%, 5-year 48.2%	No
This study	Retrospective (2 institutions)	2011–2021	39	71	20–60 Gy	1–5	27 months	1-year 100% 2-year 88.3% 3-year 73.6%	1-year 94.4%, 2-year 94.4%, 3-year 83.7%	One grade 3 radiation pneumonitis

*fx* Fractions;* SBRT* Stereotactic body radiation therapy

The dose-response relationship of SBRT for pulmonary metastases of sarcoma is uncertain. The dose-fractionation regimens implemented in previous studies, which are summarized in Table 5, include regimens with low BED. However, good LCpT rates were reported in these studies. As previously mentioned, the dose-fractionation regimens used in this study had a wide range of BED_10_ (40–180 Gy), but there was no statistically significant difference in LCpT by BED_10_ groups. One of the previously mentioned studies also investigated local control according to BED. Frakulli et al. [21] showed that there was no difference in LCpT according to BED_3_ and BED_10_. On the contrary, some studies, which analyzed both intra- and extra-thoracic metastatic lesions, showed a statistically significant dose-response relationship. Greto et al. [29] analyzed 77 metastatic lesions from 40 patients treated by SBRT. 60% of the lesions were pulmonary metastatic lesions. They showed that BED_5_ > 150 Gy was associated with improved LCpT in the multivariate analysis. Spałek et al. [15] reported a large retrospective data consisted of 233 metastatic lesions from 141 patients. 57.1% of treated lesions were pulmonary metastatic lesions. In the univariate analysis, higher equivalent dose in 2-Gy fractions (EQD2), which is a different expression of BED, was associated with increased LCpT. They did not perform multivariate analysis with EQD2 as EQD2 was strongly associated with sites of metastatic lesions. The dose-fractionation relationship of SBRT for pulmonary metastases of sarcoma cannot be concluded from the current study. However, it should be noted that a significantly higher portion of tumors in the low BED_10_ group remained stable disease at the last follow-up than in the high BED_10_ group (34.5% vs. 7.1%). This can be interpreted as high BED_10_ being needed for a durable tumor response. On the other hand, SBRT with low BED_10_ would also be utilized when the expected prognosis is poor and the indication is palliation. The dose-fractionation regimen should be decided appropriately with consideration of the indication of SBRT and potential dose-response relationship.

Although patients with pulmonary metastatic lesions may benefit from SBRT, most patients in this study experienced other metastatic events, as seen in low rates of PPFR (3-year 22.7%) and APFR (3-year 12.9%). A high OS rate (3-year 83.7%) was observed. However, considering that 29 (74.4%) patients still had disease at the last follow-up and the OS rates reported in previous literature, OS events in this study may have been underreported. In this study, oligoprogression (vs. oligometastasis), multiple pulmonary lesions at recurrence, and simultaneous extrathoracic metastasis were associated with worse prognosis. It is important to evaluate the indication for SBRT and the extent of metastatic disease when estimating treatment outcomes for patients with a limited number of pulmonary metastatic lesions from sarcoma. On the other hand, there was no prognostic significance of palliation (vs. oligometastasis) as an indication for SBRT in this study. Patient selection factors could influence this outcome. Patients who underwent palliative lung SBRT had numerous metastatic lesions that were not amenable for systemic therapy, but these patients tended to have disease with slow progression.

This study has several limitations. First, the size of the patient cohort in this study was small, which could result in low statistical power. Second, there were patient selection factors due to the retrospective nature of the study. Patients included in this study had either limited numbers of metastatic lesions or multiple metastatic disease with slow progression. These patients may have better prognosis than typical patients with metastatic sarcoma. Third, interpreting chest CT scans for response and recurrence evaluation was challenging due to radiation pneumonitis, and bias may have influenced the decision. Finally, this study evaluated sarcoma as a whole, but sarcoma is a very heterogeneous disease depending on the histologic subtype and primary site. The patients were referred to the radiation oncology department at various stages of metastatic disease, making the heterogeneity of the cohort more intensive. Nevertheless, this study reported valuable clinical data on SBRT for pulmonary metastasis of sarcoma, as studies in this category of disease are scarce and have small sample sizes. Furthermore, this study provided several insights, including the potential for durable response and prognostic factors of metastatic sarcoma.

In conclusion, SBRT can provide good local control for pulmonary metastasis of sarcoma. Some patients with oligometastasis may have durable clinical response with long-term disease-free intervals by applying SBRT. Although the dose-response relationship of SBRT is uncertain, patients who may benefit from achieving a durable response may need SBRT with a high BED_10_. Consideration of SBRT indication and disease extent may be needed as these factors may significantly influence the prognosis.

## Supplementary Information

**Additional file 1: Table 1.** Dose-fractionation regimens used in stereotactic body radiation therapy courses

## Data Availability

The datasets used and/or analyzed during the current study are available from the corresponding author on reasonable request.

## Abbreviations

APFR: Any progression-free rate
BED_10_: Biologically effective dose with an α/β ratio of 10
CI: Confidence interval
CT: Computed tomography
EQD2: Equivalent dose in 2-Gy fractions
HR: Hazard ratio
ITV: Internal target volume
LCpP: Local control per patient
LCpT: Local control per tumor
OS: Overall survival
PPFR: Pulmonary progression-free rate
PTV: Planning target volume
SBRT: Stereotactic body radiation therapy
