# Supplementary material for: Treatment outcomes of stereotactic body radiation therapy for pulmonary metastasis from sarcoma: a multicenter, retrospective study
Source: Radiat Oncol. 2023 Apr 15;18:68. doi: 10.1186/s13014-023-02255-y (PMC10105948; doi:10.1186/s13014-023-02255-y)
Supplement: Supplementary file 1 — Additional file 1: Table 1. Dose-fractionation regimens used in stereotactic body radiation therapy courses [file 13014_2023_2255_MOESM1_ESM.docx]

**Supplementary Table 1. Dose-fractionation regimens used in stereotactic body radiation therapy courses**

| **Regimen** | **Number of tumors (N = 71)** | **BED_10_ (Gy)** | **BED_4_ (Gy)** |
| --- | --- | --- | --- |
| 60 Gy in 3 fractions | 2 (2.8%) | 180.0 | 360.0 |
| 46 Gy in 2 fractions | 1 (1.4%) | 151.8 | 301.5 |
| 54 Gy in 3 fractions | 1 (1.4%) | 151.2 | 297.0 |
| 60 Gy in 4 fractions | 11 (15.5%) | 150.0 | 285.0 |
| 54 Gy in 4 fractions | 8 (11.3%) | 126.9 | 236.3 |
| 52 Gy in 4 fractions | 2 (2.8%) | 119.6 | 221.0 |
| 45 Gy in 3 fractions | 1 (1.4%) | 112.5 | 213.8 |
| 48 Gy in 4 fractions | 13 (18.3%) | 105.6 | 192.0 |
| 50 Gy in 5 fractions | 3 (4.2%) | 100.0 | 175.0 |
| 40.5 Gy in 3 fractions | 1 (1.4%) | 95.2 | 177.2 |
| 26 Gy in 1 fraction | 3 (4.2%) | 93.6 | 195.0 |
| 44 Gy in 4 fractions | 4 (5.6%) | 92.4 | 165.0 |
| 25 Gy in 1 fraction | 1 (1.4%) | 87.5 | 181.3 |
| 40 Gy in 4 fractions | 4 (5.6%) | 80.0 | 140.0 |
| 30 Gy in 2 fractions | 2 (2.8%) | 75.0 | 142.5 |
| 40 Gy in 5 fractions | 1 (1.4%) | 72.0 | 120.0 |
| 30 Gy in 3 fractions | 9 (12.7%) | 60.0 | 105.0 |
| 32 Gy in 4 fractions | 2 (2.8%) | 57.6 | 96.0 |
| 20 Gy in 2 fractions | 2 (2.8%) | 40.0 | 70.0 |

Abbreviation: BED_4_, biologically effective dose with an α/β ratio of 4; BED10, biologically effective dose with an α/β ratio of 10.
